# Supplementary material for: Important Difference between Occupational Hazard Exposure among Shift Workers and Other Workers; Comparing Workplace before and after 1980
Source: Int J Environ Res Public Health. 2020 Oct 15;17(20):7495. doi: 10.3390/ijerph17207495 (PMC7602472; doi:10.3390/ijerph17207495)
Supplement: Supplementary file 1 [file ijerph-17-07495-s001.pdf]

Supplementary Material

Table S1. Number and percentage of missing values for outcome variables.

	n Valid	n Missing	% Missing
Exposed to noise	118,577	836	0.7
Exposed to cold	117,515	1898	1.6
Exposed to hot	117,447	1,966	1.6
Exposed to dust	116,814	2,599	2.2
Exposed to chemical/fumes	116,289	3,124	2.6
Exposed to smoke	117,177	2,236	1.9
Exposed to asbestos	102,745	16,668	14.0
Exposed to paints/ thinners/glues	116,660	2,753	2.3
Exposed to pesticides	115,737	3,676	3.1
Exposed to diesel exhaust	116,598	2,815	2.4

Table S2. Prevalence ratio of workplace hazards among shift workers compared to other workers (reference). Sensitivity analyses.

	Job ended before 1980 n = 59,692						Job ended after 1980 n = 59,721					
**Exposure**	**% Shift workers**	**% Other workers**	**PR**	**95% CI Lower**	**Upper**	**P value**	**% Shit workers**	**% Other workers**	**PR**	**95% CI Lower**	**Upper**	**P value**
Noise	64.0	39.6	1.59	1.55	1.62	0.00E+00	70.3	43.4	1.58	1.56	1.61	0.00E+00
Cold	35.3	27.0	1.25	1.20	1.29	0.00E+00	43.7	29.7	1.39	1.35	1.43	0.00E+00
Hot	56.7	36.2	1.48	1.44	1.52	0.00E+00	64.3	43.4	1.43	1.40	1.46	0.00E+00
Dust	29.8	25.2	1.14	1.09	1.18	3.58E-09	32.2	23.9	1.27	1.23	1.32	0.00E+00
Chemical/fumes	26.2	16.1	1.50	1.43	1.57	0.00E+00	29.5	13.8	1.93	1.85	2.00	0.00E+00
Smoke	48.4	53.2	0.88	0.86	0.91	0.00E+00	45.5	40.3	1.06	1.03	1.08	1.32E-05
Asbestos	13.6	7.1	1.51	1.40	1.63	0.00E+00	13.1	5.9	1.73	1.62	1.86	0.00E+00
Paints/ thinners/ glues	13.2	10.2	1.16	1.08	1.24	5.18E-05	15.2	10.5	1.27	1.20	1.34	5.55E-16
Pesticides	2.0	1.8	0.91	0.75	1.12	0.380	2.8	2.0	1.17	1.01	1.35	0.039
Diesel exhaust	16.5	6.8	1.99	1.87	2.13	0.00E+00	20.1	6.5	2.40	2.28	2.54	0.00E+00
Exposed to ≥2hazards	64.8	49.5	1.26	1.21	1.31	0.00E+00	71.0	50.2	1.37	1.33	1.42	0.00E+00

Analysis were adjusted for gender, ethnicity, education, age, neuroticism and stratified by time. PR: Prevalence ratio; CI: confidence interval

Table S3. Prevalence ratio of workplace hazards after 1980 compared to before 1980 (reference). Sensitivity analyses.

	Shift workers n = 17,239						Other workers n = 102,174					
**Exposure**	**% Before 1980**	**% After 1980**	**PR**	**95% CI Lower**	**95% CI Upper**	**P value**	**% Before 1980**	**% After 1980**	**PR**	**95% CI Lower**	**95% CI Upper**	**P value**
Noise	64.0	70.3	1.05	1.03	1.08	6.07E-05	39.6	43.4	1.13	1.11	1.15	0.00E+00
Cold	35.3	43.7	1.16	1.12	1.22	2.61E-12	27.0	29.7	1.09	1.06	1.11	2.98E-11
Hot	56.7	64.3	1.09	1.05	1.12	7.58E-08	36.2	43.4	1.19	1.16	1.21	0.00E+00
Dust	29.8	32.2	1.06	1.01	1.12	0.013	25.2	23.9	0.93	0.91	0.96	3.28E-07
Chemical/fumes	26.2	29.5	1.12	1.05	1.20	0.001	16.1	13.8	0.77	0.74	0.80	0.00E+00
Smoke	48.4	45.5	0.95	0.92	0.99	0.007	53.2	40.3	0.79	0.78	0.81	0.00E+00
Asbestos	13.6	13.1	1.11	1.01	1.21	0.029	7.1	5.9	0.92	0.87	0.97	0.003
Paints/ thinners/ glues	13.2	15.2	1.07	0.97	1.19	0.149	10.2	10.5	0.95	0.91	1.00	0.046
Pesticides	2.0	2.8	1.57	1.23	2.00	3.35E-04	1.8	2.0	1.00	0.90	1.12	0.993
Diesel exhaust	16.5	20.1	1.17	1.09	1.26	2.73E-05	6.8	6.5	0.87	0.82	0.92	1.43E-06
Exposed to ≥2hazards	64.8	71.0	1.04	1.01	1.07	0.005	49.5	50.2	1.03	1.02	1.05	1.46E-04

Analysis were adjusted for gender, ethnicity, education, age, neuroticism and stratified by shift pattern. PR: Prevalence ratio; CI: confidence interval
